# The immunomodulatory effect of sugammadex *in vitro* and after total hip arthroplasty: A randomised controlled pilot and retrospective cohort study

**DOI:** 10.1097/EJA.0000000000002132

**Published:** 2025-02-14

**Authors:** Veerle Bijkerk, Lotte M.C. Jacobs, Jetze Visser, Esmee V. van Helden, Christiaan Keijzer, Leonie S. Helder, Kim I. Albers, Michiel C. Warlé

**Affiliations:** From the Radboud University Medical Center, Department of Surgery (VB, LMCJ, MCW), Radboud University Medical Center, Department of Anesthesiology (VB, EvH, CK, KIA), Radboud University Medical Center, Department of Orthopedics (JV), Radboud University Medical Center, Department of Internal Medicine, Nijmegen, The Netherlands (LSH)

## Abstract

**BACKGROUND:**

Postoperative immunosuppression is a well known phenomenon associated with infectious complications. Peri-operative immune dysregulation is likely induced by surgical damage and anaesthetics, but remains far from comprehensively characterised. To address this, the effects of individual drugs on immune function must be explored. Sugammadex, a cyclodextrin that encapsulates rocuronium, also binds other drugs and structures and may influence the inflammatory response.

**OBJECTIVE:**

Investigate the potential immunomodulatory effect of sugammadex

**DESIGN:**

An in-vitro experiment, randomised controlled pilot study and retrospective cohort study

**SETTING:**

Tertiary teaching hospital

**PATIENTS:**

Twelve healthy donors, 20 adults undergoing total hip arthroplasty and 1000 major abdominal surgery patients

**INTERVENTION:**

*In vitro:* isolated peripheral blood mononuclear cells were exposed to sugammadex and rocuronium before stimulation with *Escherichia coli* lipopolysaccharides (LPS).

*Pilot study:* patients undergoing total hip arthroplasty under single shot spinal anaesthesia randomised to sugammadex (8 mg kg^-1^) or placebo at the end of surgery.

**MAIN OUTCOME MEASURE:**

*In vitro:* TNF, IL-1β and IL-6 production capacity.

*Pilot study:* Ex-vivo cytokine production capacity after whole blood stimulation with LPS.

*Retrospective cohort:* sugammadex as a predictor of postoperative infectious complications

**RESULTS:**

*In vitro*: rocuronium suppressed TNF and IL-1β production capacity. Higher doses of sugammadex (100 and 1000 μg ml^-1^; 100 μg ml^-1^ corresponds to plasma concentration reached upon 8 mg kg^-1^ sugammadex) restored suppression of TNF and IL-1β.

*Pilot study:* no differences in ex-vivo cytokine production capacity between the sugammadex and placebo group at the end of surgery or on postoperative day 1.

*Retrospective cohort study*: no association between sugammadex and postoperative infectious complications (OR = 1.000, 95% CI 0.998 to 1.002).

**CONCLUSION:**

Sugammadex preserved cytokine production capacity of TNF and IL-1β *in vitro*. The clinical pilot study and retrospective cohort study revealed no early postoperative immunomodulatory effects for sugammadex in the clinically used dosing range.

**TRIAL REGISTRATION:**

clinicaltrials.gov identifier: NCT05723406 and NCT05244655.

**KEY POINTS**

- The effects of individual anaesthetic agents on postoperative immunosuppression are far from comprehensively characterised.
- In an in-vitro experiment, sugammadex preserved TNF and IL-1β production capacity.
- In a clinical pilot study and retrospective cohort study, this association could not be confirmed.
- There is no evidence of a postoperative immunomodulatory effect of sugammadex in the clinically used dosing range.

## Introduction

Postoperative infections continue to be one of the most common complications after surgery, with a prevalence ranging from 4 to 10%.^1–3^ They exert a significant impact on both patients and healthcare systems due to their association with increased mortality, morbidity and prolonged hospitalisation.^1,4^ Although infection rates after arthroplasties are lower, periprosthetic joint infection is a devastating complication, accounting for a great invalidity.^5–7^ Hence, it is of the utmost importance to reduce the risk of this complication.

Reduction of postoperative infections and faster recovery have been achieved with improvements in procedure-related factors, such as aseptic working conditions and the implementation of fast-track surgery. However, the challenge of eliminating postoperative infections remains.^8^ In this light, recent focus has shifted towards comprehending the physiological and pathophysiological responses of the immune system to surgery. Surgical trauma induces the release of damage-associated molecular patterns (DAMPs) out of injured cells. DAMPS bind to pattern recognition receptors on antigen-presenting cells, initiating the cascade of cytokines release.^9–11^ tumour necrosis factor (TNF) and interleukin (IL)-1β are elevated shortly after trauma or surgery and activate other pro-inflammatory mediators such as IL-6, but also anti-inflammatory mediators such as IL-10.^11,12^ Dysregulation of immune homeostasis due to concomitant pro-inflammatory and anti-inflammatory responses is believed to be a key factor in postoperative infectious complications.^13,14^ In trauma patients, increased plasma levels of TNF and a decline in ex-vivo cytokine responsiveness of TNF and IL-1β is associated with multiple organ dysfunction syndromes.^15,16^ In abdominal surgery patients, an association was found between infectious complications and decline in postoperative ex-vivo cytokine production capacity of TNF and IL-1β.^17^

Methods to counteract this immunosuppressive state are hard to elucidate. Reducing surgical trauma, by for example laparoscopy, is thought to limit the inflammatory and anti-inflammatory responses.^18^ In addition, different intra-operative anaesthetic agents are believed to exert varying effects on the immune response, but the effects of individual drugs are far from comprehensively characterised and need to be explored. However, separating or controlling for those potential immune-influencing factors is challenging in peri-operative clinical trials.

Sugammadex is a modified γ-cyclodextrin that reverses neuromuscular blockade by encapsulating nondepolarising neuromuscular blocking agents (NMBAs). However, sugammadex can bind other cellular structures, albeit with a lower affinity.^19^ For instance, in previous research in animal models, it was found that sugammadex may exert a limiting effect on neuro-inflammatory processes and has an anti-inflammatory role.^20^ It is therefore possible that sugammadex could have extended effects and may influence the inflammatory response.

Therefore, this study examined the potential positive immunomodulatory impact of sugammadex. An initial assessment of the effect of sugammadex was made through an in-vitro study. A subsequent clinical pilot study investigated the influence of sugammadex at the end of total hip arthroplasty (THA) under neuraxial anaesthesia on postoperative immune suppression. A retrospective cohort study was performed to evaluate the effect of sugammadex administration on 30-days postoperative complications after major abdominal surgery.

## Materials and methods

First, an in-vitro experiment was conducted to assess the immunomodulatory effects of isolated sugammadex exposure and combined with rocuronium. Subsequently to the in-vitro experiment, a clinical pilot study was conducted to further assess the effect of sugammadex. The pilot study compromised a single-centre, randomised controlled pilot study performed at the Radboud University Medical Center (Nijmegen, the Netherlands). Permission was granted by both the Medical Research Ethics Committee ‘METC Oost-Nederland’ (NL82808.091.22, 25/01/2023) and the competent authority. All individuals provided written informed consent prior to study participation. This manuscript adheres to the applicable CONSORT guidelines. Lastly, a post hoc analysis of a retrospective cohort study was performed to evaluate the findings of the clinical pilot study.

### In-vitro experiment

In the in-vitro experiment, isolated peripheral blood mononuclear cells (PBMCs) from healthy donors were exposed to sugammadex and rocuronium prior to stimulation with *Escherichia coli* (*E. coli*) lipopolysaccharides (LPS). Afterwards, cytokine production capacity was quantified to assess the functionality of immune cells.

Buffy coats were obtained out of blood samples of 12 healthy volunteers after written informed consent (Sanquin Blood Bank, Nijmegen, The Netherlands). PBMCs were isolated from buffy coats using density gradient centrifugation over Ficoll-Paque (GE Healthcare, Uppsala, Sweden). Subsequently, they were washed three times with phosphate-buffered saline at 4°C. Afterwards, PBMCs were suspended in cell culture medium [Roswell Park Memorial Institute (RPMI) 1640 Dutch Modified (Gibco, Thermo Fisher Scientific, Waltham, MA, USA)] supplemented with gentamycin 50 μg ml^-1^, pyruvate 1 mmol l^-1^ and GlutaMAX 2 mmol l^-1^. Hereafter, suspended PBMCs were cultured in a 96-well round bottom plate with 5 x 10^5^ per well, and they were incubated one hour (37°C, 5% CO_2_) with only culture medium or rocuronium (0.5, 5, 15, 50 and 100 μmol l^-1^). Afterwards, another incubation period of 1 h followed with culture medium or sugammadex (10, 100 and 1000 μg/ml), resulting in cells incubated with only culture medium (negative control), rocuronium, sugammadex or a combination. After incubation, cells were stimulated with 10 ng ml^-1^*E. coli* (serotype O55:B5; Sigma Aldrich, St Louis, Missouri, USA) for 24 h to be able to assess the functionality of immune cells. After 24 h of stimulation, samples were centrifuged (1400 RPM at room temperature for 8 min) and supernatants were stored at -20°C until analysis.

To assess immune cell functionality after incubation with sugammadex and rocuronium, cytokine concentrations were determined in the supernatants [TNF, IL-6, IL-1β]. This was done batchwise using ELISAs according to the manufacturer's instructions (R&D systems, Minneapolis, Minnesota, USA).

### Clinical pilot study

In the clinical pilot study, sugammadex was administered at the end of THA under neuraxial anaesthesia, to study the effect on the postoperative innate immune function in the absence of rocuronium. As this study was a pilot study, no sample size calculation was performed. It was planned to include 20 patients, based on comparable studies.^13,16,21,22^

#### Participants

To be eligible for study participation, patients had to be 18 years or older and scheduled for primary THA under neuraxial anaesthesia. Exclusion criteria were insufficient control of the Dutch language to understand the patient information and fill out the questionnaires, contraindication to the use of sugammadex or suxamethonium, deficiency of vitamin K dependent clotting factors or coagulopathy, severe renal (creatinine clearance < 30 ml min^-1^) or liver disease (Child-Pugh classification C), and chronic use of psychotropic drugs, NSAIDs, steroids or immunosuppressive drugs. In addition, we excluded women who were pregnant, currently breastfeeding or in childbearing potential without the use of adequate contraception. Lastly, we excluded patients unable to make informed choices or consent due to impaired intellectual capacity. Patients were screened for eligibility by their treating orthopaedic surgeon.

#### Randomisation and blinding

Shortly before surgery, patients were randomly assigned to the intervention or control group. The intervention group received sugammadex 8 mg kg^-1^ at the end of surgery and the control group received a placebo (NaCl 0.9%) instead. Computer-generated randomisation was performed by Castor EDC's (Castor Electronic Data Capture^23^) in a 1 : 1 fashion with variable block size randomisation to conceal allocation. No stratification was performed. Patients and all personnel were blinded for treatment allocation, including the anaesthesiologist and their assistant. Study medication was administered by an unblinded research physician who did not have a role in outcome assessment to ensure blinding of the patient and personnel. As no emergency occurred, no unblinding was required.

#### Anaesthesia and surgery

Neuraxial anaesthesia, namely single shot spinal anaesthesia, was obtained with bupivacaine 0.5% 10 to 20 mg combined with sufentanil. If preferred by the patient, additional sedation could be obtained by propofol infusion of 1.5 to 4.5 mg kg^-1^ h^-1^. According to local protocol, patients received paracetamol, ascorbic acid and pregabalin as premedication. In addition, esketamine was administered intra-operatively. Administration of dexamethasone was avoided, to prevent influence on postoperative immune outcome measurements. The THA was performed via posterolateral approach and with cemented prostheses. At the end of the surgery, the study medication was administered during skin closure.

#### Outcomes

The primary outcome of this trial was postoperative innate immune function as reflected by the ex-vivo cytokine production capacity upon whole blood stimulation. Blood samples were obtained at three timepoints: before surgery, after administration of study medication and at postoperative day 1. Whole blood was stimulated *ex vivo* with LPS of the *E. coli* as in the in-vitro experiment and described elsewhere.^24^ In brief, 0.5 ml of whole blood was added to either a tube with 2 ml culture medium (negative control) or a tube with culture medium supplemented with 12.5 ng ml^-1^*E. coli* LPS (final concentration 10 ng ml^-1^, serotype O55:B5; Sigma Aldrich). Tubes were incubated for 24 h (37°C with 5% CO_2_) and afterwards centrifuged (10 min at 3800 RPM at room temperature). Post-LPS stimulated supernatants were stored at -80°C until analysis. To evaluate the immune system's functionality, cytokine concentrations (TNF, IL-6, IL-1β, IL-10) were determined batchwise using Human Bio-Techne R&D ELISAs according to the manufacturer's instructions (R&D systems, Minneapolis, Minnesota, USA, catalogue numbers DY206, DY201, DY210 and DY217B).

Secondary outcomes included further assessment of innate immune function by measurement of circulating inflammatory cytokines in the obtained blood samples of the three different timepoints. Obtained blood samples in lithium heparin and EDTA blood tubes were centrifuged (3800 RPM at room temperature for 10 min), and EDTA samples were centrifuged again [16 000 relative centrifugal force (RCF) at room temperature for 10 min]. Afterwards, plasma samples were stored at -80°C until analysis. Plasma concentrations of pro-inflammatory and anti-inflammatory cytokines (TNF, IL-6, IL-10) were measured batchwise using a Luminex assay according to the manufacturer's instructions (Milliplex; Millipore, Billerica, Massachusetts, USA).

Additional secondary outcomes consisted of health-related quality of life, pain perception and postoperative complications. Health-related quality of life was assessed by the validated Quality of Recovery-40 (QoR-40) questionnaires,^25^ completed by patients pre-operatively and at postoperative day 1. In addition, pain perception was assessed by a numeric rating scale (NRS; 0 to 10) at the post anaesthesia care unit (PACU) and at postoperative day 1. Complications were registered as part of standard care up to 30 days postoperative according to the Clavien-Dindo classification.^26^ Infectious complications were scored according to the criteria of the StEP-COMPAC group initiative.^27^

### Retrospective cohort analysis

To evaluate the findings of the clinical pilot study, we performed a posthoc analysis from a recently published retrospective cohort study on early postoperative pain and 30-day complications after major abdominal surgery.^28^ This study compromises 1000 patients undergoing surgery between January 2014 and December 2020 in Radboud University Medical Center. Included procedures were oesophageal surgery, liver surgery, pancreatic surgery and cytoreductive surgery and hyperthermic intraperitoneal chemotherapy (HIPEC). For all patients 30-day postoperative infectious were scored along the Centers for Disease Control definitions of healthcare-associated infections. Full methodical explanation of the data collection can be found in the original study.^28^ In addition to the published data, the database includes data on sugammadex administration for each patient. A potential association between administration of sugammadex and postoperative infectious complications has been investigated.

### Statistical analysis

Cytokine production capacities of the in-vitro experiment were compared to control by Friedman tests and Dunn's post hoc test for nonnormal distribution. In the clinical pilot study on THA, differences in cytokine production capacity and plasma cytokines between groups were compared using Mann–Whitney *U* test and differences over time with Friedman tests and Dunn's post hoc test. Variation in baseline characteristics among the two groups were compared using standardised differences. Calculated according to the sample size, variables with ASD more than 0.66 were considered imbalanced.^29^ For additional secondary outcomes, the Student's *t*-test was used for continuous data in case of normality, and for nonnormally distributed data, the Mann–Whitney *U* test was used. Categorical data were compared by χ^2^ test. *P* values less than 0.05 were considered significant. Due to the exploratory nature of this study on a small scale, characterised by a small sample size, correction for multiple testing was not applied. All analyses were on an intention-to-treat base and no imputation was performed for missing data.

For the posthoc retrospective cohort analysis, a multivariable logistic regression was used to investigate whether there was an association between sugammadex administration and 30-days postoperative infectious complications. Preceding this analysis, potential explanatory variables were first tested one by one and included in the multivariable logistic regression model in case of a *P* value less than 0.05. Sugammadex was added to the multivariable logistic regression model to evaluate its effect in relation to other explanatory variables. Statistical analysis was performed using SPSS software (SPSS V.27.0, IBM, Released 2020, Armonk, New York, USA).

## Results

### In-vitro experiment

**Fig. 1:**
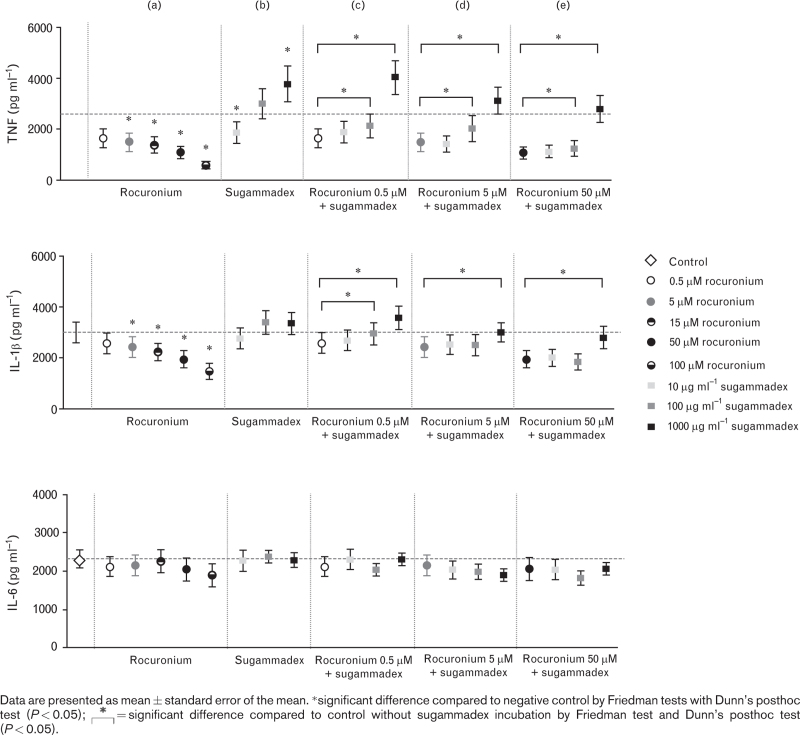
In-vitro production capacity of tumour necrosis factor (TNF), interleukin (IL)-1β and IL-6 upon PBMCs (*n* = 12) stimulation with *Escherichia coli* lipopolysaccharide after incubation with rocuronium 0.5 to 100 μmol l^-1^ (a), sugammadex 10 to 1000 μg ml^-1^ (b) or a combination (c-e).

The in-vitro experiment demonstrated that incubation with solely rocuronium before LPS stimulation led to a dose-dependent suppression of cytokine production capacity as compared to the negative control, as shown in Fig. 1a. This reduction was significant for TNF and IL-1β for the concentration of rocuronium of 5, 15, 50 and 50 μmol l^-1^, and at the highest concentrations also for IL-6. Rocuronium did not affect cell viability as assessed by cytotoxicity assay.

Incubation with only sugammadex before LPS stimulation showed an interesting dose-dependent trend for TNF. In lower doses, it reduced TNF production capacity, whereas in higher doses, it increased TNF production capacity (Fig. 1b). Sugammadex alone did not seem to influence production capacity of IL-1β and IL-6. Further analysis revealed that for combination of rocuronium and sugammadex, high doses of sugammadex (100 and 1000 μg ml^-1^) counteracted the inhibition of TNF and IL-1β production by rocuronium (Fig. 1c–e).

### Clinical pilot study

**Fig. 2:**
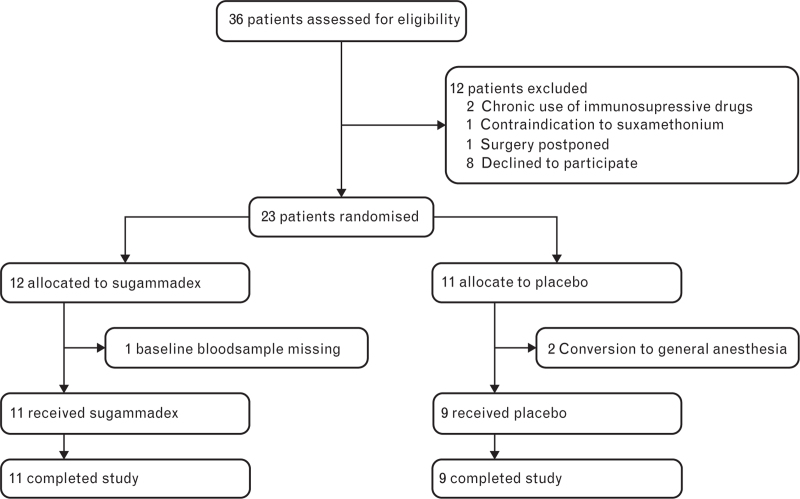
CONSORT flow diagram clinical pilot study.

The clinical pilot study was conducted between March 2023 and October 2023, and 36 patients were assessed for eligibility. Twenty-three patients were randomised instead of the initially planned 20 inclusions, as three patients were excluded and replaced after randomisation. Specifically, neuraxial anaesthesia was deemed not possible in the operating room for two patients, and blood samples could not be obtained from one patient. The remaining 20 patients completed the study (Fig. 2). Baseline characteristics and intra-operative parameters were similar between the two groups and are presented in Table 1.

**Table 1:** Baseline characteristics

	Sugammadex (*n* = 11)	Control (*n* = 9)	*P*	Standardised difference
Patient characteristics				
Age	48.8 ± 17.2	61.7 ± 18.9	0.129	0.71
Female/Male sex	4 / 7	4 / 5	0.714	0.17
BMI	28.4 ± 5.0	27.5 ± 6.1	0.724	0.16
ASA classification			0.723	0.37
I	5 (45.5)	3 (33.3)		
II	3 (27.3)	2 (22.2)		
III	3 (27.3)	4 (44.4)		
History of smoking	3 (30.0)	5 (55.5)	0.384	0.66
Packyears	7.5 ± 4.3	16.9 ± 12.7	0.275	0.99
Side of hip replacement, L/R	4 / 7	4 / 5	0.714	0.17
Comorbidities				
Cardiovascular disease	8 (72.7)	5 (55.6)	0.423	0.36
Pulmonary disease	7 (63.6)	6 (66.7)	0.888	0.06
Renal insufficiency	0 (0)	0 (0)	/	0.00
Neurological disease	1 (9.1)	3 (33.3)	0.183	0.62
Liver insufficiency	0 (0.0	1 (11.1)	0.257	0.50
Diabetes mellitus	0 (0)	0 (0)	/	0.00
Intra-operative parameters				
Duration of surgery (min)	110 ± 23	102 ± 15	0.400	0.38
Blood loss (mL)	518 ± 290	429 ± 175	0.407	0.37
Propofol (mg)	487 ± 407	404 ± 279	0.609	0.24
Esketamine (mg)	18.9 ± 13.7	13.5 ± 11.4	0.363	0.42
Bupivacaine (mg)	14.5 ± 1.1	13.7 ± 1.3	0.160	0.67
Sufentanil (μg)	1.8 ± 2.4	3.9 ± 5.2	0.254	0.53
Ephedrine (mg)	9.1 ± 9.7	0.8 ± 1.8	0.019	1.18
Norepinephrine (μg)	79 ± 132	127 ± 138	0.445	0.35

Data are given as *n* (italic) (%) or mean ± SD. ASA, American Society of Anaesthesiologists classification; SD, standard deviation.

#### Innate immune function

**Fig. 3:**
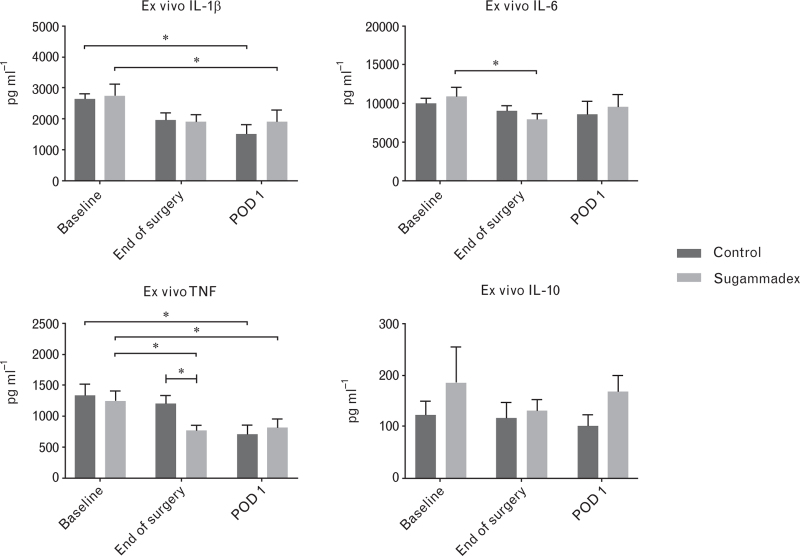
Cytokine production capacity upon ex-vivo whole blood stimulation with *Escherichia coli* lipopolysaccharide in patients receiving sugammadex (*n* = 11) or placebo (*n* = 9) at the end of total hip arthroplasty.

Overall, there was no difference in cytokine production capacity upon stimulation over time between the sugammadex and placebo group (Fig. 3). In both groups, production capacity of TNF and IL-1β significantly decreased at POD 1 compared to baseline, confirming the immunosuppression after surgery. At the end of surgery, TNF seemed to be better preserved in the placebo group compared to the sugammadex group (sugammadex 768 ± 318 pg ml^-1^ versus placebo 1196 ± 412 pg ml^-1^, *P* = 0.025). This difference disappeared at POD 1. As for IL-10 and IL-6, no trend could be observed overtime.

Data are presented as mean ± standard error of the mean. ∗*P* < 0.05 compared to baseline determined by Friedman tests with Dunn's post hoc test and compared between groups by Mann–Whitney *U* test. IL, interleukin; POD, postoperative day; TNF, tumour necrosis factor.

**Fig. 4:**
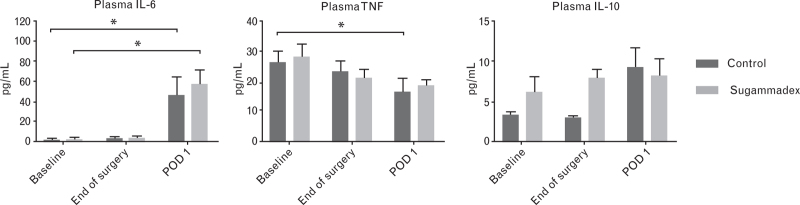
Plasma cytokine levels at baseline, the end of surgery and on postoperative day 1 (POD 1) in patients receiving sugammadex (*n* = 11) or placebo (*n* = 9) at the end of total hip arthroplasty.

In addition, there were no differences between the sugammadex and placebo group regarding plasma cytokines at any of the timepoints (Fig. 4). For both groups, a significant increase of IL-6 could be seen at POD 1 as compared to baseline.

Data are presented as mean ± standard error of the mean. ^∗^*P* < 0.05 compared to baseline determined by Friedman tests with Dunn's post hoc test. IL, interleukin; TNF, tumour necrosis factor.

#### Clinical outcomes

Overall, no statistically significant differences were observed between the intervention and the control group regarding clinical outcomes, as presented in Table 2. The mean quality of recovery-40 at postoperative day 1 in the sugammadex group compared to the placebo group was 166 ± 13 versus 160 ± 13 points (*P* = 0.323). Mean pain scores at postoperative day one did not differ between the sugammadex and control group (NRS at rest 4.6 ± 2.5 versus 4.3 ± 2.3, *P* = 0.783), (NRS upon movement 5.6 ± 2.4 versus 6.0 ± 1.7, *P* = 0.639). Five complications were registered in this study, of which four were classified as Clavien-Dindo category I without needing any treatment. One patient in the control group received antibiotic treatment for a superficial wound infection.

**Table 2:** Clinical outcomes

		Sugammadex (*n* = 11)	Control (*n* = 9)	*P*
Postoperative outcomes				
PACU stay (min)		61 [39 to 88]	57 [41 to 99]	0.909
QoR-40 POD 1		166 ± 13	160 ± 13	0.323
Time until first mobilisation (h)		22 [18 to 24]	18 [17 to 24]	0.647
Hospital stay (days)		2 [1 to 2]	2 [1 to 2]	0.684
Readmission		0 (0)	0 (0)	/
Postoperative complications		3 (27.3)	2 (33.3)	0.795
Pain				
Baseline	NRS at rest	4.6 ± 1.8	4.7 ± 2.8	0.908
	NRS upon movement	6.0 ± 2.1	6.7 ± 2.2	0.501
PACU	NRS in rest	0.0 ± 0.0	0.2 ± 0.6	0.500
	Pain medication received yes	3 ± 27.3	4 ± 44.4	0.423
POD 1	NRS in rest	4.6 ± 2.5	4.3 ± 2.3	0.783
	NRS upon movement	5.6 ± 2.4	6.0 ± 1.7	0.639

Data are given as median [IQR] mean ± SD or *n* (italic) (%). IQR, interquartile range; NRS, numeric rating scale; PACU, post anaesthesia care unit; POD 1, postoperative day 1; QoR-40, Quality of Recovery-40; SD, standard deviation.

### Retrospective cohort analysis

Out of the 1000 patients in the retrospective cohort study, 186 patients received sugammadex, with a mean dose of 170 ± 77.5 mg. A total of 289 patients encountered infectious complications. Univariate logistic regression analysis revealed that sugammadex administration was no significant predictor of 30-day postoperative infectious complications (OR = 1.000, 95% CI 0.998 to 1.002, *P* = 0.959) (Table 3). This was confirmed in the multivariable regression analysis. In addition, rocuronium was no significant predictor of 30-day postoperative infectious complications either (OR = 1.002, 95% CI 1.000 to 1.005, *P* = 0.069).

**Table 3:** Logistic regression model 30-days infectious complications

	Univariate regression			Multivariable regression		
Variable	Odds ratio	95% confidence interval	*P*	Odds ratio	95% confidence interval	P-value
Age	1.022	1.009 to 1.036	0.001	1.013	0.999 to 1.028	0.067
Sex (1 = male)	1.580	1.189 to 2.099	0.002	1.377	1.014 to 1.868	0.040
ASA classification	1.735	1.386 to 2.173	<0.001	1.719	1.353 to 2.184	<0.001
Duration of surgery (min)	1.002	1.001 to 1.003	<0.001	1.002	1.000 to 1.003	0.014
Type of surgery						
HIPEC	0.917	0.619 to 1.358	0.665			
Oesophageal	1.812	1.340 to 2.452	<0.001	1.391	0.967 to 2.001	0.075
Liver	0.386	0.274 to 0.544	<0.001	0.538	0.340 to 0.852	0.008
Pancreas	1.376	1.029 to 1.840	0.031			
Open procedure	1.188	0.873 to 1.617	0.274			
Sugammadex administration (mg)	1.000	0.998 to 1.002	0.959	1.000	0.998 to 1.002	0.774
Rocuronium administration (mg)	1.002	1.000 to 1.005	0.069			

ASA, American Society of Anaesthesiologists classification; HIPEC, hyperthermic intraperitoneal chemotherapy.

## Discussion

This study investigated the effect of sugammadex on the innate immune system. The in-vitro experiment indicated that sugammadex preserved the production capacity of TNF and IL-1β in contradiction to the immunosuppressive effect of rocuronium. However, we could not confirm any postoperative immunomodulatory effect of sugammadex in the clinical dosing range in patients undergoing THA.

Sugammadex is a modified γ-cyclodextrin specifically designed and introduced for its ability to encapsulate NMBAs to reverse neuromuscular blockade.^30^ This ability has led to a substantially faster recovery from neuromuscular blockade, enabling prompt adaptation of sugammadex in regular practice. Despite its primary function of encapsulating NMBAs, sugammadex exhibits an ability to bind or encapsulate other structures, albeit with a lower affinity.^31^ In the presence of NMBAs, displacement interactions are rare. However, under conditions of excessive sugammadex, elevated levels of free form sugammadex, or in the absence of NMBAs, interactions with other drugs or molecules are possible, potentially causing immunomodulatory effects.^19^ For example, due to structural similarities with NMBAs, steroids can also bind sugammadex, and lower steroid concentrations have been reported after sugammadex administration.^32,33^ Nevertheless, the true extent of these reactions and their clinical relevance remain debated.^33–35^

To explore these potential immunomodulatory actions of sugammadex in greater detail, we conducted a study of both fundamental research, a clinical pilot study and a retrospective cohort study. The initial in-vitro experiment yielded evidence for an immunomodulatory effect of sugammadex. Varying doses of 10, 100 and 1000 μg ml^-1^ sugammadex were used, corresponding to plasma concentrations expected after administrations of 2, 8 and 64–96 mg kg^-1^ sugammadex.^36,37^ Despite the promising results of the in-vitro experiment, the immunomodulatory effect was not observed in the pilot study. A dose of 8 mg kg^-1^ was administered, which is considered relatively high in daily practice, but potentially insufficient to elicit an observable effect. In the in-vitro experiment, the highest concentration was associated with a significant boost in cytokine production capacity, but this dose far exceeded the typical clinical dosage. Moreover, the nature of the in-vitro stimulation experiment imposed a fixed concentration of sugammadex on the cells for 24 h, which does not precisely mimic plasma concentrations over time in patients as the half-life time of sugammadex is approximately 2 h.^37^ In addition, in-vivo plasma concentrations are dynamic due to pharmacodynamic processes, creating challenges in directly translating clinically administered doses to in-vitro concentrations. Although these complexities stress the limitations in translating basic research to clinical outcomes, the combination of both approaches in this study provides insight into the potential effects of sugammadex on the immune system.

The findings of the clinical pilot study reveal a notable reduction of IL-1β and TNF production capacity on postoperative day 1 compared to baseline. This indicates a state of immunosuppression, consistent with previous findings of postoperative immune dysregulation.^13,38,39^ It is thought that, similar as in trauma, tissue injury initiates a concomitant pro-inflammatory and anti-inflammatory reaction through danger-associated molecular patterns and pattern recognition receptors.^40^ This is supported by the increased plasma IL-6 levels observed in the pilot study at postoperative day 1. Strategies to counteract this immune dysregulation are of interest. However, this study shows that it seems unlikely that sugammadex has the capability to address this dysregulation at doses used in daily practice.

In addition to the studied impact of sugammadex, the in-vitro experiment provided insights in the potential influence of rocuronium on immune function. Notably, it revealed a dose-dependent reduction of the TNF and IL-1β production capacity following rocuronium incubation. Administration of a single dose of rocuronium of 0.9 mg kg^-1^ leads to peak plasma concentrations of around 12 000 ng ml^-1^ (equivalent to 22.6 μmol l^-1^) and steady-state plasma concentrations around 1000 to 2000 ng ml^-1^ (equivalent to 1.9 to 3.8 μmol l^-1^) during continuous infusion.^41,42^ The decrease of cytokine production capacity may be explained by the competitive binding of rocuronium to nicotinic acetylcholine receptors (nAch). Those receptors are also present on immune cells, including macrophages, dendritic cells and lymphocytes. For instance, the α7 nACh subunit plays an important role in the cholinergic anti-inflammatory pathway that regulates immune cell proliferation, T-cell differentiation, antigen presentation and cytokine production.^43^ Competitive binding by rocuronium may thereby induce immune dysregulation. However, as we did not study the effect of rocuronium in a clinical study, it is crucial to approach these results with same caution as the sugammadex outcomes. Further research is warranted to explore this potential issue.

By integrating an in-vitro experiment with a clinical pilot study and a retrospective cohort study, this study enhances our comprehension of the subject matter. The difficulties and limitations of the in-vitro experiment interpretations are countered by the direct answer on the immunomodulatory effect of sugammadex in the clinical settings. An additional strength of this study lies in the elimination of rocuronium's influence in the pilot study. This allows us to conclude that the lack of observed effect of sugammadex is not merely a concomitant effect of NMBAs. Nevertheless, a limitation of the study is that in the in-vitro experiment, LPS stimulation is performed on PBMCs and in the clinical pilot on whole blood, which limits direct comparison of absolute numbers. In addition, investigating PMBCs *in vitro* did not embody all the components of the inflammatory response, although PBMCs are crucial in the early stages of the innate immune response. In the combined in-vitro and clinical study, we focused on the affected capacity of mononuclear cells to respond to a pathogen and to observe the difference in trends. In our opinion, those limitations do not hamper the ability to study the difference in trends between the in-vitro experiment and clinical study. Another limitation is that we merely focused on sugammadex and rocuronium and did not study other neuromuscular blocking agents or reversal agents, so no broad conclusion can be formed on muscle relaxation management. Given the small sample size and exploratory nature of this study, we opted not to correct for multiple testing. This decision increases the risk of false-positive results. However, this concern is primarily applicable to the in-vitro experiment, the only section yielding positive results. The combination of both experiments leads us to challenge those positive results and conclude that it seems unlikely that sugammadex has clinically relevant immunomodulatory effects. The effect of rocuronium seen in the in-vitro experiment only highlights the need for further study into anaesthetics’ impact on postoperative immune responses.

## Conclusion

This combined study of an in-vitro experiment, a clinical pilot study and a retrospective cohort study showed that high doses of sugammadex preserved production capacity of TNF and IL-1β in-vitro. The clinical pilot study revealed no postoperative immunomodulatory effects regarding cytokine production capacity for sugammadex in the clinically used dosing range in patients undergoing THA. This was confirmed in the retrospective cohort study as no effect of sugammadex on postoperative infectious complications were seen in patients undergoing major abdominal surgery.
